# Fragility of the Schrödinger Cat in thermal environments

**DOI:** 10.1038/s41598-023-45701-3

**Published:** 2023-10-31

**Authors:** Sandip Bera, Kenny L. S. Yip, Sajeev John

**Affiliations:** Department of Physics, University of Toronto, 60 St. George Street, Toronto, ON M5S 1A7 Canada

**Keywords:** Bose-Einstein condensates, Statistical physics

## Abstract

We describe the decoherence instability of Schrödinger Cat states in the two-site Bose-Hubbard model with an attractive on-site interaction between particles. For *N* particles with onsite attractive energy *U* and hopping amplitude between sites *t*, Cat states exist for $u\equiv \frac{UN}{2t}<-1$ at zero temperature. However, they are increasingly unstable to small thermal fluctuations as the Cat itself is increasingly well-defined and its components become well-separated. For any given $u<-1$, the decoherence temperature becomes smaller for large *N*. The loss of off-diagonal coherence peaks in the equilibrium density matrix is dominated by the thermal admixture of the first excited state of the many-body system with its ground state. Particle number fluctuations, described in the grand canonical ensemble also reduce coherence, but to a lesser degree than thermal fluctuations. The full density matrix of the Schrödinger Cat is obtained by exact numerical diagonalization of the many-body Hamiltonian and a narrow regime in the parameter space of the particle number, temperature, and *U*/*t* is identified where small Cat states may survive decoherence in a physical environment.

## Introduction

The accessibility and stability of entangled quantum many-body states^1–5^ is of fundamental importance in quantum science and technology^6–9^. Quantum stability^10^ involves the maintenance of quantum coherence when the system is in contact with a physical environment, with which it can exchange energy. While a large variety of exotic quantum states^11–15^ can be considered theoretically, only a small subset of such states may be accessible, stable, and amenable to predetermined external control and manipulation.

Controllable, macroscopic, quantum coherence is well-known in certain many-body systems near their ground states. These include low-temperature systems such as superfluids^16^ and superconductors^17^. On mesoscopic size scales and shorter time frames, many-body quantum coherence has been studied in cold atomic gases^18–22^ and exciton-polariton Bose-Einstein condensates^23–28^. Notwithstanding these remarkable discoveries, the realization of coherent quantum superposition states involving large numbers of material particles has remained extremely elusive^29,30^. The stability and controllability of quantum states involving many qubits^31^, exhibiting quantum superposition and entanglement, is central to quantum information science and quantum computing^32–36^.

In this article, we consider the two-site, attractive, Bose-Hubbard model, with *N* particles, in contact with a thermal environment, described by the canonical and grand canonical ensembles. Ho and Ciobanu^37^ proposed that, at zero temperature, this system can exhibit a ground state involving a coherent quantum superposition of many particles on one site with many particles on the other site. When these two components of the many-body ground state are sufficiently well distinguished, the state is referred to as a Schrödinger Cat^38–41^. The response of such states, at zero temperature, to small perturbations has been a subject of considerable interest in mathematical physics^42,43^. At finite temperature, we evaluate the full density matrix of this system by the exact numerical solution of all the eigenvalues and eigenfunctions of this interacting many-body system. We identify the narrow range of system parameters, including onsite attractive energy *U*, hopping amplitude between sites *t*, particle number *N*, and temperature *T*, for which the entangled Schrödinger Cat state retains quantum coherence between its two components. This coherence is measured by the magnitude of the off-diagonal peaks in the system density matrix. We show that the more distinguished the components of the Cat state, the more rapid the loss of quantum coherence between those components with small thermal fluctuations. Moreover, the larger the number of particles constituting the Cat state, the more susceptible it is to decoherence in a physical environment.

The article is organized as follows: In section “Many-body Hamiltonian”, we introduce the many-body, two-site, Bose-Hubbard model and map it to an approximate, continuum, Sturm-Liouville, differential equation. We show that the ground state and low-lying excited states can be accurately described, under suitable circumstances, by a WKB approximation describing tunnelling between two minima of a double well potential. In section “Numerical results”, we provide an exact numerical solution for the energy eigenvalues and eigenstates of the original many-body Hamiltonian. This leads to an exact evaluation of the system density matrix and off-diagonal coherence, as a function of temperature, in the canonical ensemble. This reveals the fragility of Cat states in the presence of small thermal fluctuations. In section “Particle number fluctuations”, we evaluate the full thermal density matrix, in the presence of an external particle reservoir, using the grand canonical ensemble. In section “Discussion and conclusions”, we discuss possible physical realizations and provide our conclusions.

## Many-body Hamiltonian

The two-site Bose-Hubbard model describes the behavior of bosonic particles confined to two sites, labeled as site *a* and site *b*. The model is characterized by the Hamiltonian:

1 $$\begin{aligned} \mathcal {H}=-t \left( a^{\dagger }b+b^{\dagger }a \right) + \frac{U}{2}\Big [n_{a}(n_{a}-1)+n_{b}(n_{b}-1)\Big ], \end{aligned}$$

where *t* represents the hopping amplitude between the two sites, $a^{\dagger }$ and $b^{\dagger }$ are the creation operators for bosons at sites *a* and *b*, respectively, *a* and *b* are the corresponding annihilation operators, and $n_a=a^{\dagger }a$ and $n_b=b^{\dagger }b$ are the associated number operators. $N=n_a +n_b$ is the total number operator. These operators satisfy the commutation relations $\left[ a,a^{\dagger }\right] =1$, $\left[ b,b^{\dagger }\right] =1$, and $\left[ a,b^{\dagger }\right] =[a,b]=0$.

The first term of the Hamiltonian $-t(a^{\dagger }b+b^{\dagger }a)$ describes the hopping of bosons between the two sites. The hopping strength *t* determines the rate at which bosons can move from one site to the other. The overall hopping energy scales with $-tN$. The second term $\frac{U}{2}\Big [n_{a}(n_{a}-1)+n_{b}(n_{b}-1)\Big ]$ accounts for the on-site interaction between bosons. *U* is the amplitude of on-site interaction. The sign of *U* determines the nature of the interaction: positive values correspond to repulsive interactions, where bosons tend to avoid each other, while negative values indicate attractive interactions, where bosons tend to cluster together. The overall interaction energy scales with $UN^2$. For the hopping term and the on-site interaction term to exert comparable influence on the bosonic system, we require *tN* to be comparable to $UN^2$. In other words, *UN*/*t* is a dimensionless measure of the on-site interaction strength that properly scales with *N*.

### Analytical model

The boson number operator commutes with the Hamiltonian $[\mathcal{H},N]=0$. Thus, energy eigenstates can be chosen to have a definite total number of bosons. We diagonalize the Hamiltonian in equation (1) using the orthogonal basis of Fock states^37,44^: $|\Psi \rangle =\sum _{l}\Psi _{l}|l\rangle$, where $|l\rangle$ represents the normalized quantum state $|l,N-l\rangle$ describing *l* bosons on site *a* and $N-l$ bosons on site *b*. Here, *l* takes values from 0 to *N*. The coefficients $\Psi _{l}$ are yet-to-be-determined wavefunction amplitudes. After substituting this ansatz into the Schrödinger equation corresponding to the Hamiltonian (1), we obtain the eigenvalue equation:

2 $$\begin{aligned} E\Psi _l=-t_{l-1}\Psi _{l-1}-t_{l}\Psi _{l+1}+ V_{l}\Psi _{l}, \end{aligned}$$

where $t_{l}=t\sqrt{(N-l)(l+1)}$, $~V_{l}=\Big [ U\Big (l-\frac{N}{2}\Big )^2 +\frac{UN}{2}(\frac{N}{2}-1)\Big ]$ and *E* is the energy eigenvalue. This describes a quantum particle hopping in a one-dimensional lattice with $N+1$ sites, in a potential $V_l$ and for which the hopping amplitudes vary with location, with hopping toward either endpoint of the lattice becoming increasingly difficult. We perform numerical diagonalization of the above Hamiltonian with an orthonormal Fock state basis of $N+1$ vectors (*N* bosons), using the Mathematica software^45,46^. We discuss the exact numerical results later.

A more intuitive physical picture is obtained by mapping the above lattice problem to an effective continuum model. We show, below, that the finite difference equation (2) can be mapped to a Sturm-Liouville differential equation in a continuous variable, *y*, that ranges from 0 to *N*. We introduce the definition of first difference $\frac{\Delta \Psi }{\Delta l}|_{l+1/2} \equiv \Psi _{l+1}-\Psi _{l}$. The above eigenvalue equation (2) can then be expressed in the form of a discrete Sturm-Liouville problem: $E\Psi _{l}=-\frac{\Delta }{\Delta l}\Big [t_{l-1/2}\frac{\Delta \Psi }{\Delta l}\Big ]\Big |_{l} + V_{\text {eff}}(l)\Psi _l$, where $V_{\text {eff}}(l)=U\Big (l-\frac{N}{2}\Big )^2 -t_{l}-t_{l-1}+\frac{UN}{2}(\frac{N}{2}-1)$ is an effective lattice potential. We extend the discrete wavefunction amplitude $\Psi _{l}$ to a continuous and differentiable function $\Psi (y)$. According to the Lagrange mean value theorem, $\frac{d \Psi }{d y}(y=\zeta ) = \Psi _{l+1}-\Psi _{l}$ for some $\zeta$ in the open interval $(l,~l+1)$. Using the ansatz, $\zeta =l+1/2$, the eigenvalue equation in continuum limit becomes: $E\Psi (y)=-\frac{d}{d y}\Big [t_{y-1/2}\frac{d \Psi (y)}{d y}\Big ]+ V_{\text {eff}}(y)\Psi (y)$, where $V_{\text {eff}}(y)= U\Big (y-\frac{N}{2}\Big )^2 -t_{y}-t_{y-1}+\frac{UN}{2}(\frac{N}{2}-1)$ and $\Psi (y)$ represent the effective potential and the wavefunction, respectively, in the continuum limit, where $0 \le y \le N$. Here, $t_{y}=t\sqrt{(N-y)(y+1)}$.

For convenience, we introduce the translated variable $x \equiv y-N/2$, define over the symmetrical interval $-\frac{N}{2} \le x\le \frac{N}{2}$. The eigenvalue equation is expressed in terms of *x*:

3 $$\begin{aligned}&E\Psi (x)=-\frac{d}{d x}\Big [t_{x-1/2}\frac{d \Psi (x)}{d x}\Big ]+ V_{\text {eff}}(x)\Psi (x), \end{aligned}$$

4 $$\begin{aligned}&V_{\text {eff}}(x)= U x^2 +\frac{UN}{2}\left( \frac{N}{2}-1 \right) -t_{x}-t_{x-1}, \end{aligned}$$

where $t_{x}=t\sqrt{\frac{N}{2} \left( \frac{N}{2}+1\right) -x(x+1)}$ can be interpreted as the reciprocal of the position-dependent effective mass in the continuum model.

### Effective potential in the continuum model

We identify the critical transition by the change of concavity of $V_{\text {eff}}(x)$, and map the second order differential equation (3) to that of a simple harmonic oscillator (SHO). The effective potential $V_{\text {eff}}(x)$ can be expressed in Taylor series about $x=0$:

**Figure 1:**
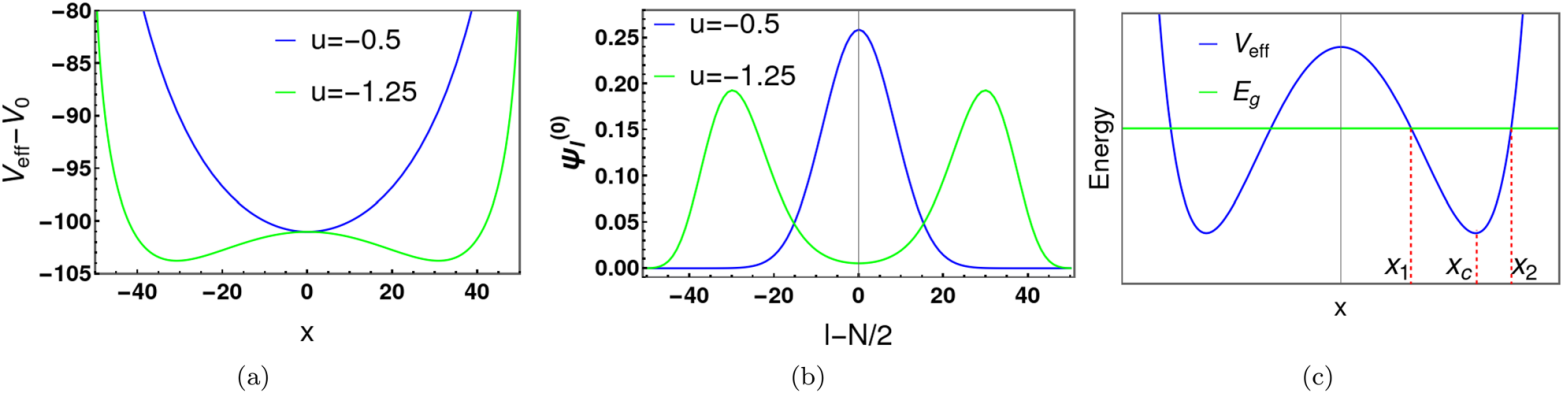
Panels (**a**) and (**b**) display the characteristics of the effective potential and the ground state wave function, respectively, for two different values of *u*, one above ($u=-\,0.5$ or $U=-\,0.01$) and one below ($u=-1.25$ or $U=-0.025$) the *u* critical value $(u=-\,1)$. Here $V_{0}=\frac{UN}{2}(\frac{N}{2}-1)$. The other parameters used for this calculation are $N=100$ and $t=1.0$. For $u=-\,0.5$, the ground state wavefunction has a single Gaussian peak. For $u<-\,1.0$, the effective potential has two local minima and the ground state wavefunction exhibits two Gaussian peaks. Panel (**c**) shows the classical turning points ($x_1$ and $x_2$) for $E=E_{g}$ (the ground state energy) and the location, $x_c$, of the potential minimum. Here, the green line and blue line denote the ground state energy and effective potential, respectively.

5 $$\begin{aligned} V_{\text {eff}}(x)&\approx -2t\sqrt{\alpha _{1}}+\frac{1}{2} \left( \frac{N^2}{2}-N\right) U+t\sqrt{\alpha _{1}}\Bigg [\frac{U}{t\sqrt{\alpha _{1}}}+\frac{1+4\alpha _{1}}{4\alpha _{1}^2}\Bigg ]x^2 +t\sqrt{\alpha _{1}}\Bigg [\frac{(1+4\alpha _{1})(5+4\alpha _{1})}{64\alpha _{1}^4}\Bigg ]x^4+ \cdots , \end{aligned}$$

where $\alpha _{1}=\frac{N}{2} \left( \frac{N}{2}+1\right)$. The coefficients of all non-vanishing terms in the series are positive, except for the $x^2$ and $x^0$ terms. The coefficient of $x^2$ vanishes when $\frac{U N}{ 2t} (\equiv u)=- \sqrt{\frac{N}{N+2}} \left[ 1 + \frac{1}{N(N+2)}\right]$.

For large *N*, the effective potential $V_{\text {eff}}(x)$ is simplified to

6 $$\begin{aligned} V_{\text {eff}}(x) \approx U x^2 + \dfrac{1}{4} U N^2 - tN \left( 1 - \dfrac{4x^2}{N^2} \right) ^{1/2} \approx \dfrac{1}{4} U N^2 - t N + \left( U + \dfrac{2t}{N} \right) x^2 + \cdots \text {.} \end{aligned}$$

Under the simplifying condition, the coefficient of $x^2$ changes its sign at $\frac{U N}{ 2t} (\equiv u)=-1$. We refer to $u=-1$ as a critical point. For $u\ge -1$, $V_{\text {eff}}(x)$ has a single minimum at $x=0$. For $u<-1$, $V_{\text {eff}}(x)$ exhibits a double well structure with a pair of local minima, symmetric about the origin. Figure 1a shows the behaviour of the effective potential for two different values of *u*. The effective potential at $u=-0.5$, which is above the critical value, is shown by the blue line. The green line for $u=-\,1.25$ is below the critical value and exhibits two separated minima.

When $u<-1$, the double well potential minima occur at $x=\pm x_c$ (see Fig. 1c). For large *N*, we set $d V_{\text {eff}}/dx(x)=0$ to determine

7 $$\begin{aligned} x_{c}\approx \frac{N}{2} \left( 1-u^{-2}\right) ^{1/2}. \end{aligned}$$

In the vicinity of the local minimum at $x_c$, the effective continuum model can be approximated as a simple harmonic oscillator of mass $m_c$ and angular frequency $\omega _c$ ($\hbar =1$):

8 $$\begin{aligned} V_{\text {eff}}(x)&\approx \left( \frac{1}{2} N^2 U+\frac{t^2}{U} \right) +(x-x_{c})^2 U\left( 1-u^2\right) . \end{aligned}$$

To complete the analogy with a SHO, we identify $m_c$ using the kinetic energy term in Eq. (3) and $\omega _c$ using the quadratic term of $V_{\text {eff}}(x)$ at $x=x_c$ in Eq. (8):

9 $$\begin{aligned}&m_c \approx \frac{1}{2t_{x_c-1/2}} \approx \frac{-U}{2t^2}, \end{aligned}$$

10 $$\begin{aligned}&\omega _c \approx 2t(u^2-1)^{1/2}. \end{aligned}$$

By direct comparison with SHO, we estimate the ground state energy $E_g \approx (1/2)\omega _c$ relative to the potential minimum $V_{\text {eff}}(x=x_c)$:

11 $$\begin{aligned} E_g=t (u^2-1)^{1/2}. \end{aligned}$$

In this analogy, the ground state solution is proportional to $\exp \Big [-(x-x_c)^2 /(2\sigma _c^2)\Big ] (\equiv \Psi _{R}^{(0)} (x))$, where the width $\sigma _{c} \approx \sqrt{1/m_c\omega _c}$ of the Gaussian peak is $\sigma _c \approx [\frac{U^2}{t^2}(u^2 -1)]^{-1/4}$. Similarly, the left side minimum has a ground state proportional to $\exp \Big [-(x+x_c)^2 /(2\sigma _c^2)\Big ] (\equiv \Psi _{L}^{(0)} (x))$. Given the non-zero tunnelling amplitude between the minima, the ground state wave function for the double well potential can be approximated by a linear combination of $\Psi _{R}^{(0)} (x)$ and $\Psi _{L}^{(0)} (x)$. The numerical solution of the lattice model (2) for the ground state wave function in the double well for $u=-\,1.25$ is shown by the green line in Fig. 1b.

Well above critical point when $u=-\,1$, the ground state wave function exhibits a single Gaussian peak at origin. Near $x=0$, the effective continuum model can be approximated as a SHO of mass $m_0$ and angular frequency $\omega _0$. Similar to the previous analysis, by Eqs. (3) and (8), we identify

12 $$\begin{aligned}&m_0 \approx \frac{1}{2t_{-1/2}} \approx \frac{1}{tN}, \end{aligned}$$

13 $$\begin{aligned}&\omega _0 \approx 2t(1+u)^{1/2}. \end{aligned}$$

This approximate ground state wave function is proportional to $\exp \Big [-x^2 /(2\sigma _0^2)\Big ]$, with $\sigma _0\approx (\frac{N}{2})^{1/2}(u+1)^{-1/4}$. The numerical solution of lattice model (2) for the ground state wave function at $u=-\,0.5$ is shown by the blue line in Fig. 1b.

A ground state in which the wave form $\Psi _{R}^{(0)}+\Psi _{L}^{(0)}$ consists of distinguishable peaks at $x=\pm x_c$ corresponds to many-body quantum state of (1) that is a superposition of a significant number of bosons on site *a* with the same significant number of bosons on site *b*. In what follows, we define a well-developed Schrödinger Cat state to be one in which the ground state probability density at $x=\pm x_c$ is at least a factor of ten larger than the probability density at $x=0$. As $u\rightarrow -1$, from below, the two components of the “Cat state” merge and the peaks at $x=\pm x_c$ becomes less discernible. On the other hand, for large negative $u<-1$, the tunnelling amplitude between the two components of the “Cat” becomes negligible and we approach an “extreme Cat” state of the form $(|N,0\rangle +|0,N\rangle )/\sqrt{2}$. While such “extreme Cat” states may appear as tantalizing possibilities at zero temperature, we show below that they are unstable to decoherence for infinitesimally small thermal fluctuation.

So far, we have presented analytical approximations describing a single minimum in $V_{\text {eff}}(x)$. We describe below an analytical approximation to the tunnelling amplitude between a pair of distinct local minima that accurately describes the ground and first excited state wavefunctions, provided we are not too close to the critical point $u=-1$.

### Approximation for ground and first excited states

We apply the WKB approximation to the continuum two-site Bose-Hubbard model to estimate the energy separation $dE_{10}$ between the ground and first excited states. For $u<-\,1$, the ground state and first excited state are even and odd superpositions, respectively, of near-Gaussian wavefunctions centered at the minima of the double well potential. This double well potential intersects the ground state energy at four different classical turning points ($\pm x_1$ and $\pm x_2$), as depicted in Fig. 1c.

The WKB approximation for the ordinary Schrödinger’s equation is modified to accommodate a position-dependent effective mass in the Sturm-Liouville problem:

14 $$\begin{aligned} E \Psi = - \epsilon ^{2} \dfrac{d}{dx} \left( t_{x - 1/2} \dfrac{d \Psi }{dx} \right) + V_{\text {eff}}(x) \Psi . \end{aligned}$$

Here, we introduce $\epsilon = 1$ to keep track of the perturbation order of the WKB approximation. We begin with an exponential asymptotic approximation^47^:

15 $$\begin{aligned} \Psi _{\text {WKB}}(x) \sim \exp \left[ \frac{1}{\epsilon } \sum _{n=0}^{\infty } \epsilon ^{n} S_{n}(x) \right] , \end{aligned}$$

where $S_{n}(x)$ is the *n*th order term of the phase function. Here, we only keep the phase function up to the first order in $\epsilon$. We substitute Eq. (15) into Eq. (14) to obtain a sequence of equations which determines $S_{0}$ and $S_{1}$:

16a $$\begin{aligned} \left( \dfrac{d S_{0}}{dx} \right) ^{2}= & {} Q(x), \end{aligned}$$

16b $$\begin{aligned} 2 t_{x - 1/2} \dfrac{d S_{0}}{dx} \dfrac{d S_{1}}{dx} + t_{x - 1/2} \dfrac{d^2 S_{0}}{dx^2} + \dfrac{d t_{x - 1/2}}{dx} \dfrac{d S_{0}}{dx}= & {} 0, \end{aligned}$$

where $Q(x) \equiv (V_{\text {eff}}(x) - E)/t_{x - 1/2}$. These equations are solved to yield:

17a $$\begin{aligned} S_{0}(x)= & {} \pm \int ^{x} dx' \left[ Q(x') \right] ^{1/2}, \end{aligned}$$

17b $$\begin{aligned} S_{1}(x)= & {} -\dfrac{1}{2} \ln t_{x-1/2} - \dfrac{1}{4} \ln Q(x). \end{aligned}$$

As a result of the position-dependent effective mass, the modified WKB wavefunction gains an additional amplitude modulation factor compared to the WKB wavefunction for the standard Schrödinger’s equation^47^:

18 $$\begin{aligned} \Psi _{\text {WKB}}(x) \sim \dfrac{A}{\left( t_{x-1/2}\right) ^{1/2} \left[ Q(x)\right] ^{1/4}} \exp \left[ \pm \int ^{x} dx' \left[ Q(x')\right] ^{1/2} \right] . \end{aligned}$$

As shown below the extra factor does not affect the energy quantization conditions.

The WKB approximation fails near a classical turning point. Nevertheless, the effective potential can be linearized in the vicinity of a turning point, and the solutions to the linearized differential equation are the Airy functions. A global approximation can be constructed by matching the asymptotic expansions of the Airy functions to the WKB approximation on either side of the turning point:

19 $$\begin{aligned} \Psi _{\text {WKB}}(x) \sim {\left\{ \begin{array}{ll} \dfrac{A}{\left( t_{x-1/2}\right) ^{1/2} \left[ Q(x)\right] ^{1/4}} \bigg \{ 2 \cos \eta \exp \left[ \int _{x}^{x_{1}} dx' \left[ Q(x')\right] ^{1/2} \right] &{} \\ \qquad \qquad \qquad \qquad + \sin \eta \exp \left[ - \int _{x}^{x_{1}} dx' \left[ Q(x')\right] ^{1/2} \right] \bigg \}, &{} \text { for } 0 \le x< x_{1}, \\ \dfrac{A}{\left( t_{x-1/2}\right) ^{1/2} |Q(x)|^{1/4}} \sin \left( \int _{x}^{x_{2}} dx' |Q(x')|^{1/2} + \dfrac{\pi }{4} \right) , &{} \text { for } x_{1}< x< x_{2}, \\ \dfrac{A}{\left( t_{x-1/2}\right) ^{1/2} \left[ Q(x)\right] ^{1/4}} \exp \left[ - \int _{x_{2}}^{x} dx' \left[ Q(x')\right] ^{1/2} \right] , &{} \text { for } x_{2} < x, \end{array}\right. } \end{aligned}$$

where $\eta = \int _{x_{1}}^{x_{2}} dx' |Q(x')|^{1/2}$ is a phase angle over the classically allowed region $x_{1}< x < x_{2}$. The standard connection formulae apply because the effective mass is regular and positive at the classical turning points. Over the classically forbidden region, $0 \le x < x_{1}$, there is a factor of 2 with the $\cos \eta$ term, because of the different prefactors in the asymptotic expansions of the exponentially decaying Airy function $\text {Ai}(x)$ of the first kind and exponentially increasing Airy function $\text {Bi}(x)$ the second kind.

The WKB approximation for $x<0$ is determined by parity. For the even-parity ground state, $\Psi _{\text {WKB}}(-x) = \Psi _{\text {WKB}}(x)$. For the odd-parity first excited state, $\Psi _{\text {WKB}}(-x) = - \Psi _{\text {WKB}}(x)$. These imply the following quantization conditions: $d\Psi _{\text {WKB}}/dx (x=0) = 0$ for even parity states and $\Psi _{\text {WKB}} (x=0) = 0$ for odd parity states. Hence, by Eq. (19), we obtain

20 $$\begin{aligned} \tan \eta = \pm 2 e^{\kappa }, \end{aligned}$$

where $\kappa = \int _{-x_{1}}^{x_{1}} dx' |Q(x')|^{1/2}$ is a phase angle in the non-classical region. Here, $+$ and $-$ represent even and odd parity states, respectively. The calculations are simplified by observing that $t_{x-1/2}$ and *Q*(*x*) are even functions of *x*, so that their derivatives vanish at $x=0$.

Finally, we estimate the energy separation between the ground and first excited states, in the weak tunnelling limit ($\kappa>>1$) between the two minima of the double well potential. For $u<-1$, using the SHO approximation developed in Eqs. (9) and (10), we express the phase angle $\eta$ in term of the eigenenergy *E*:

21 $$\begin{aligned} \eta \approx \int _{x_{1}}^{x_2} dx^{\prime } \Big [ 2m_{c} \Big ( E-\frac{1}{2}m_c \omega _{c}^2 (x^{\prime }-x_c)^2 \Big )\Big ]^{1/2} =\frac{E\pi }{\omega _c}. \end{aligned}$$

where $\pm x_{1}$ and $\pm x_{2}$ are the turning points. In the weak tunnelling limit ($\kappa>>1$), the solutions of (20) occur near the poles of the tangent function $\eta _{n} \approx (n+1/2)\pi$, where $n=0,1,2,\cdots$. For the ground and low-lying excited states, we can write $\eta _{n}= (n+1/2)\pi +\delta _n$, where $\delta _n<<1$. Therefore, $\tan \eta _{n}=-\cot \delta _{n}\approx -1/\delta _{n}=\pm 2e^{\kappa }$, or

22 $$\begin{aligned} \eta _{n}\approx (n+1/2)\pi \mp \frac{1}{2}e^{-\kappa }. \end{aligned}$$

Combining Eqs. (21) and (22), we determine the energy spacing $dE_{10}$ between the ground and first excited states ($n=0$):

23 $$\begin{aligned} dE_{10}\approx \frac{\omega _c}{\pi }\Big [\frac{1}{2}e^{-\kappa }- \left( - \frac{1}{2}e^{-\kappa }\right) \Big ]=\frac{2}{\pi } E_{g} e^{-\kappa }. \end{aligned}$$

In the exponent, $\kappa$, the energy parameter can be approximated by that of the ground state.

**Figure 2:**
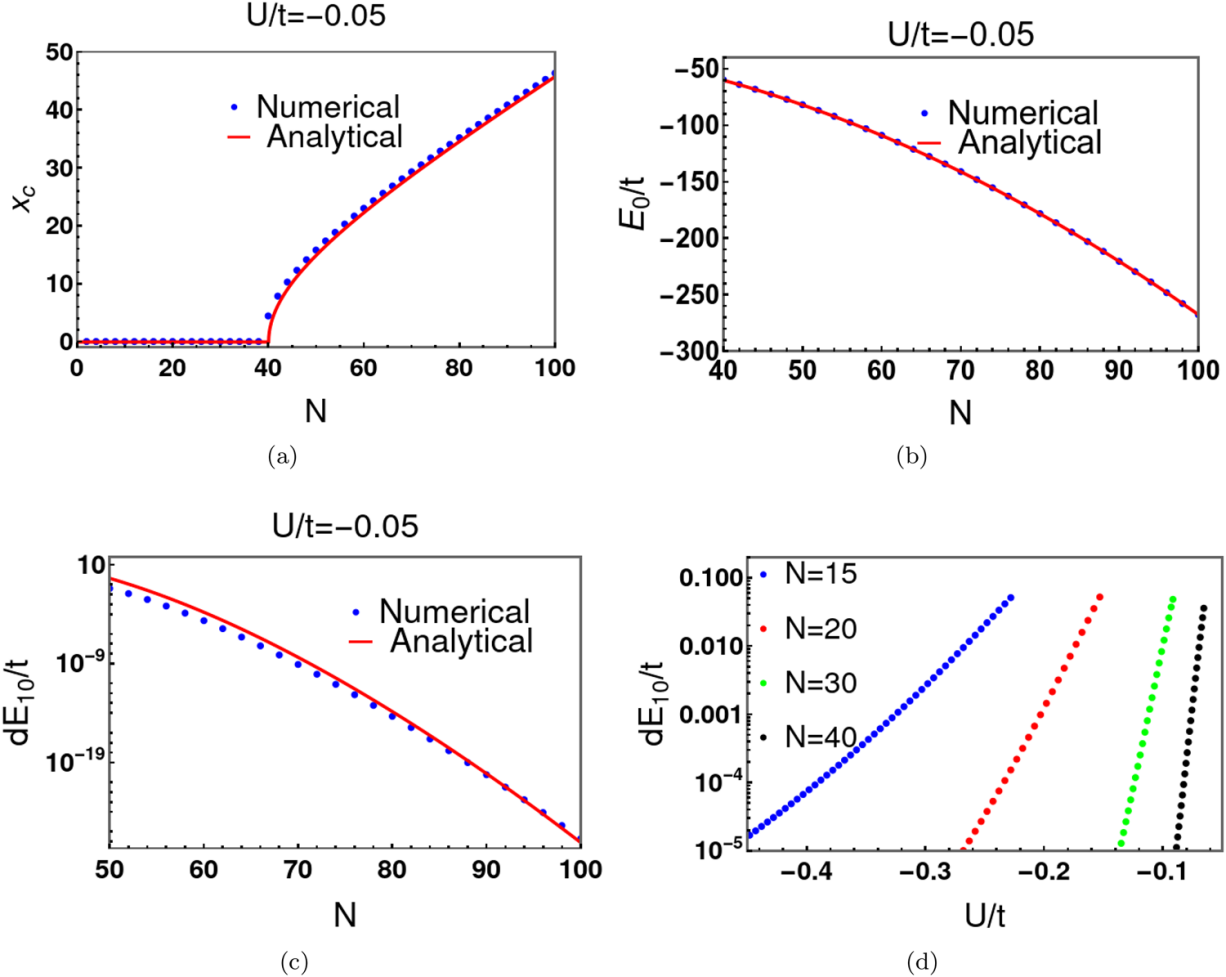
Panel (**a**) shows the behavior of the location of the potential minimum, $x_c$, with *N* at $U/t=-\,0.05$. Here, the blue dots represent the exact numerical solution of Eq. (2) and the solid red line is the approximate WKB analytical solution given in Eq. (7). Clearly, the WKB approximation provides an excellent estimate. For $U/t=-\,0.05$, a double well potential occurs if $N>40$. In panel (**b**), we compare the precise numerical results (blue dots) of Eq. (2) with the approximate WKB solutions (solid red lines) for the ground state energy $E_0$ of the original eigenvalue equations (2) and (3). Panel (**c**) depicts the energy difference between the ground and first excited states $dE_{10}$ provided by Eqs. (11) and (23). Panel (**d**) shows behaviour of $dE_{10}$ with *U*/*t* for different values of *N*. The decay of $dE_{10}$ with *U*/*t* is much faster for large *N* than for small *N*. Here, all points are obtained from exact numerical solution of Eq. (2).

## Numerical results

We have discussed the analytical expressions of $x_c$ and $E_g$ using a continuum approximation, $dE_{10}$ using the WKB approximation. We now compare our continuum analytical estimates with the exact numerical solution for the spectrum of the discrete equation (2). Solid lines in Fig. 2a depict analytical results for $x_c$. The red dots are the exact numerical results. Similar comparisons are provided for the ground state energy $E_0$ of the original Hamiltonian (1) and energy separation $dE_{10}$ between the ground state and first excited state (see Fig. 2b and c respectively). Clearly, the WKB estimates are very close to the exact numerical results. The exact numerical solution for $dE_{10}$ also indicates that $dE_{10}$ decays with *U*/*t* much faster for large *N* than for small *N* (Fig. 2d). This has important consequences for the decoherence and instability of “Cat states” consisting of a large number of particles, with the addition of extremely small thermal fluctuations.

**Figure 3:**
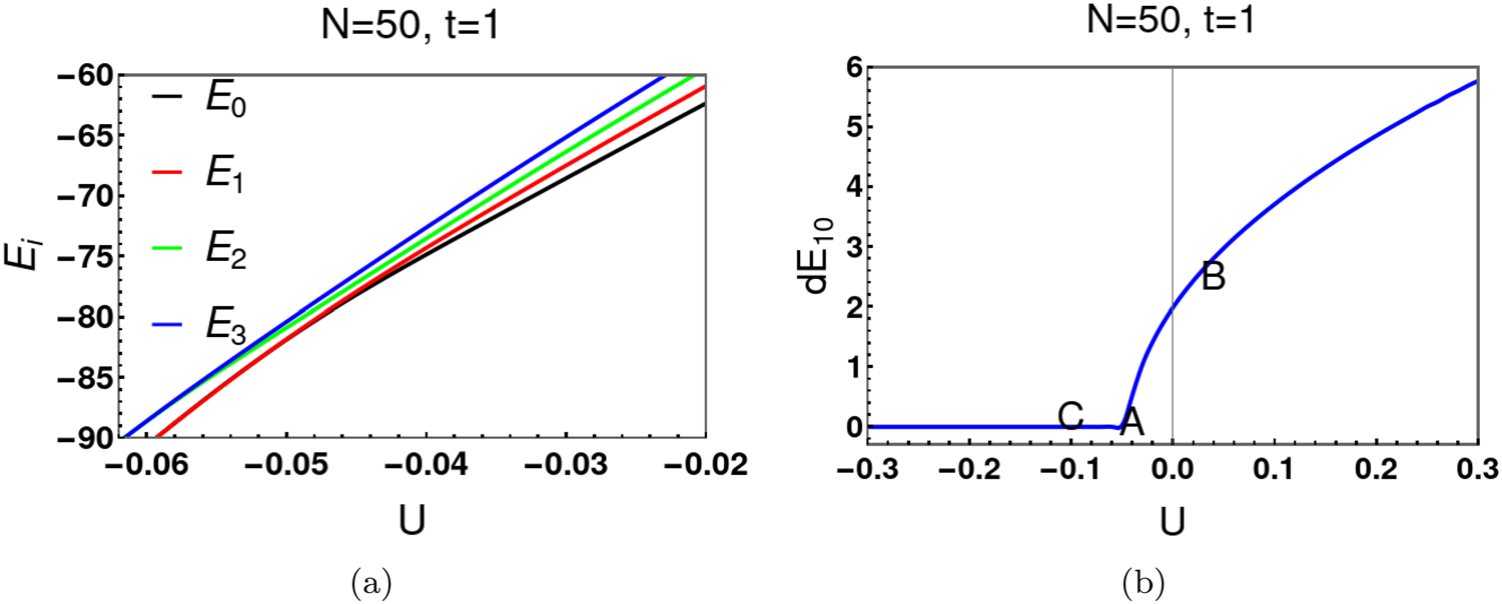
Panel (**a**) shows the behavior of several low energy states with *U* at $N=50$. In panel (**b**), the separation ($dE_{10}$) between the ground state and the first excited state is discernible for $U>0$, but drops precipitously to zero for $U<0$. For $N=50$, the critical value of *U*/*t* is $-0.04$ as indicated by *A* in panel 3b. We label another two points one above (*B*) and the other below (*C*) the critical point $u=-\,1.0$.

Figure 3a shows the energy behaviour with *U* at $N=50$. A double well potential emerges if $U/t<-0.04$, its critical value. The separation, $dE_{10}$, between the ground state energy ($E_{0}$) and the first excited energy ($E_{1}$) decreases exponentially with |*U*/*t*|, at fixed *N*, for $u<-1.0$ (see Fig. 3b). The corresponding exponential decrease of $dE_{10}$ with *N*, at fixed |*U*/*t*| is apparent in Fig. 2c. It is this behaviour that leads to exponentially rapid decoherence and fragility of Schrödinger Cat states.

**Figure 4:**
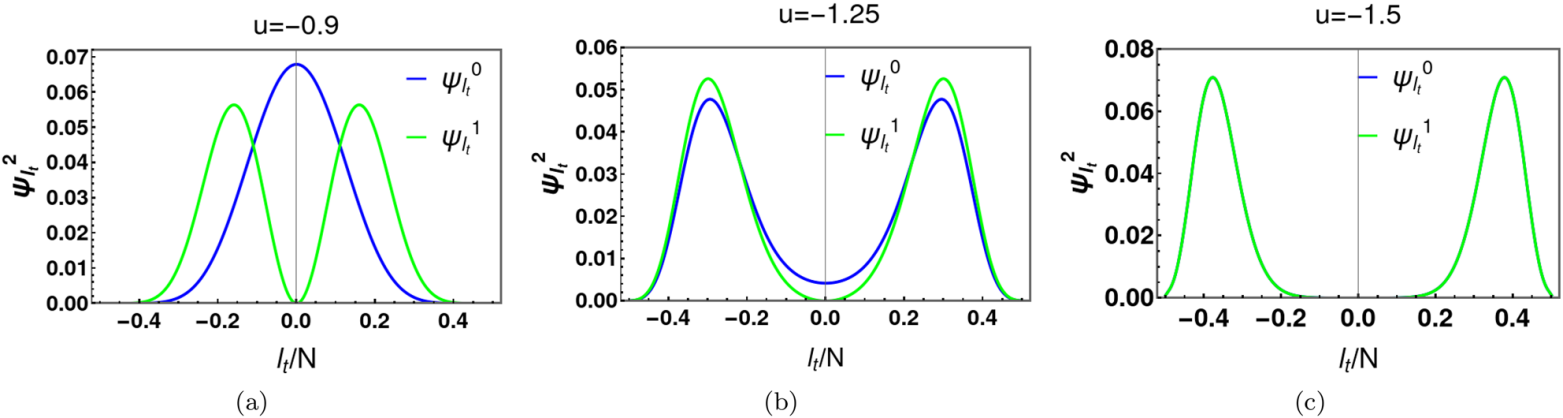
Panels (**a**–**c**) depict the square of the ground state and first excited state wave functions for three different values of *u*. $(\Psi ^{0})^2$ and $(\Psi ^{1})^2$ are markedly distinct for $u>-1$, the critical value (see (**a**)). Figure (**b**) shows the numerical results for $u=-\,1.25$. Now both $(\Psi ^{0})^2$ and $(\Psi ^{1})^2$ have a minimum point at $l_t(\equiv l-N/2)=0$, but are discernibly distinct. A further decrease in *u* to $-\,1.5$ causes distinction to become indiscernible (see (**c**)). This leads to nearly vanishing off-diagonal coherence in the density matrix at exponentially small temperature scales.

We plot (see Fig. 4) the square of the wave function, both above and below the critical point $u=-\,1.0$, for $N=50$ particles. For *N* even, it is convenient to introduce the translated discrete index $l_t\equiv l-N/2$. Figure 4a shows the square of ground state wave function and first excited wave function at $u=-0.9$. $(\Psi _{l_t}^1)^2$ is zero at $l_t=0$ and has two separate maximum around the origin, whereas $(\Psi _{l_t}^0)^2$ has one maximum at $l_t=0$. For $u<-1.0$, there are two Gaussian peaks in the ground state (see Fig. 4b), centered near the potential energy minima. The wings of these two Gaussian peaks superimpose around ${l_t}=0$. Consequently, $(\Psi _{l_t}^0)^2$ at $l_t=0$ is nonzero (see figure 4b). The first excited state of a symmetric potential is antisymmetric in nature. As a result $(\Psi _{l_t}^1)^2$ is zero at $l_t=0$. Figure 4b shows that the overlap between $(\Psi _{l_t}^0)^2$ and $(\Psi _{l_t}^1)^2$ at $u=-1.25$. The separation between the two peaks becomes more pronounced with decreasing *u* (see Fig. 4c). Moreover, the probability densities $(\Psi _{l_t}^0)^2$ and $(\Psi _{l_t}^1)^2$ become almost indistinguishable from each other. This has serious implication for the off-diagonal elements of the system’s thermal density matrix, which provides a measure of quantum coherence between the two components of the Schrödinger Cat state. For choices of *u* well below the critical point ($u=-\,1.0$), small thermal fluctuations will mix the nearly degenerate ground and first excited state. Given the close resemblance of $(\Psi _{l_t}^0)^2$ and $(\Psi _{l_t}^1)^2$, there is nearly complete phase cancellation of the off-diagonal coherence in the density matrix at temperature scales corresponding to the nearly vanishing energy scale $dE_{10}$ (see Fig. 3b).

### Region for robust Cat state

**Figure 5:**
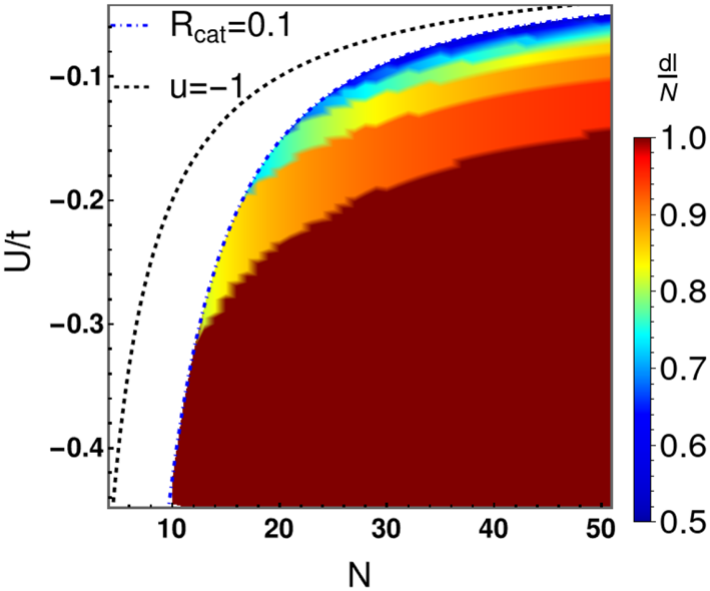
Depicted is the separation (*dl*) between the two Gaussian peaks (components of the Cat) in the ground state wave function $\Psi ^{0}$. We divided *l* by *N* to fit all results on the same scale. So, *l*/*N* ranges from 0 to $+1$. The upper dotted line represents the locus of the critical points for which $u=-\,1.0$. The lower dotted line represents the locus of points below which our condition for a well-developed Cat state, $R_{cat}=0.1$, is satisfied (see main text).

As described above, the ground state has two Gaussian peaks for $u<-\,1.0$. The separation (*dl*) between the two Gaussian peaks depends on *U*/*t* and *N*. Figure 5 shows the behaviour of *dl*/*N* in the $U/t-N$ plane. The upper dotted line represents the critical condition $u=-\,1.0$ for the onset of the double well potential. The lower dashed line demarks the region where a well developed “Cat state” appears. This is defined by the condition that $R_{cat}\equiv \frac{|\Psi ^{0}(l_{t}=0)|^2}{|\Psi ^{0}( l_{\text {peak}})|^2}\le 0.1$, where $\pm l_{\text {peak}}$ represent the locations of the Gaussian peaks in the double-well potential. Above this line, the Gaussian probability densities overlap noticeably. Below this line, the probability densities associated with the two components of the Cat are well-distinguished. When $dl/N \rightarrow 1$, the ground state approaches an extreme Cat state (NOON state) of the form $(|N,0\rangle +|0,N\rangle )/\sqrt{2}$.

### Density matrix and decoherence temperature

**Figure 6:**
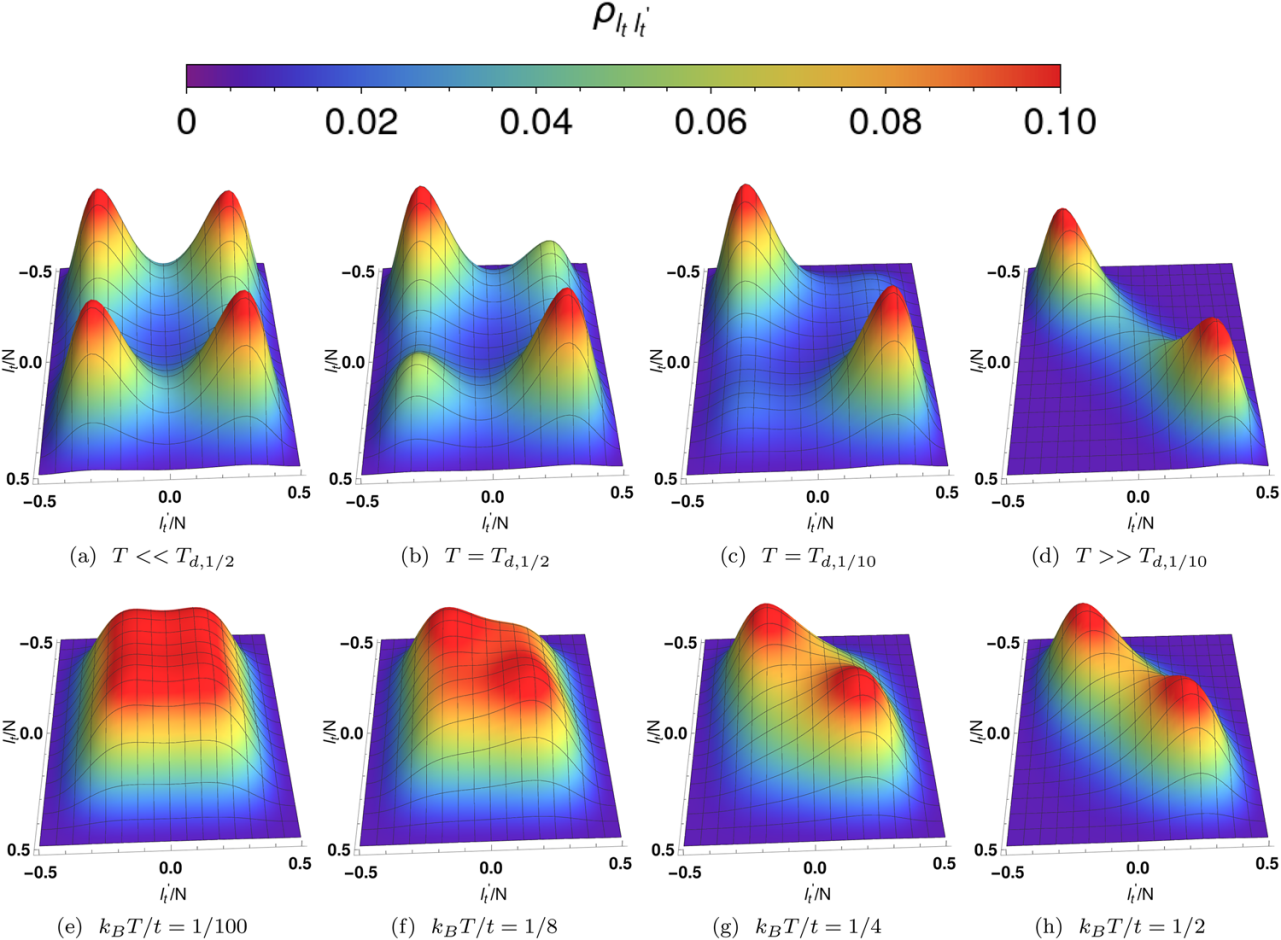
Panels (**a**–**d**) depict the density matrices of well-developed ($$R_{cat}= 0.09$$) Cat states at four distinct temperatures for $$U/t=-\,0.05, N=50$$ ($$u=-\,1.25$$). In panel (**a**), the density matrix contains four nearly equal-amplitude peaks at very low temperatures $$T<<T_{d,1/2}$$. Panels (**b**) and (**c**) depict density matrices for $$T=T_{d,1/2}$$ and $$T=T_{d,1/10}$$, respectively. Here, $$T_{d,R}$$ is defined as the temperature where the ratio between the diagonal peak to the off-diagonal peak is *R*. The height of the off-diagonal peaks becomes smaller as the temperature increases. In panel (**d**), $$T>>T_{d,1/10}$$ and the amplitude of the diagonal peaks is noticeably diminished as the peaks broaden and merge. Here, the Cat components are less separated and coherence is lost. Panels (**e**–**h**) exhibit the density matrix for four distinct temperatures at $$U/t=-\,0.044, N=50$$ ($$u=-\,1.11$$). Since *u* is close to the critical point ($$u=-\,1.0$$), the Cat components are not well separated ($$R_{cat} =0.98 $$) and the states are referred to as weak Cat states.

We now consider the finite-temperature density matrix for fixed *N*. The off-diagonal coherence peaks of this matrix provide a measure of the stability of Cat states to thermal fluctuations. The density matrix of this system is given by: ${\varvec{\rho }}=\sum _{i=0}^{N} P(E_i) |\Psi ^{(i)}\rangle \langle \Psi ^{(i)}|$, where $P(E_i)=\frac{1}{Z_{N}} \exp (-\beta E_i)$ is the probability distribution, $\beta =\frac{1}{k_{B}T}$, *T* is the temperature and $Z_N$ is the partition function in canonical ensemble. For *N* bosons, the Hilbert space has $N+1$ basis states. Therefore, the density matrix is an $(N+1)\times (N+1)$ matrix. The $l,l^{\prime }$th element of the matrix is given by: $\rho _{l,l^{\prime }}=\frac{1}{Z_{N}}\sum _{i=0}^{N} e^{-\beta E_{i}}\Psi ^{(i)}_{l}\Psi ^{(i)}_{l^\prime }$. Here both *l* and $l^{\prime }$ range from 0 to *N*. At low temperatures ($k_{B}T<dE_{10}$), the density matrix exhibits four peaks-two diagonal and two off-diagonal, dominated the ground state probability $P(E_0)$. As the temperature increases, more excited states contribute to the density matrix. The resulting phase cancellation from the excited states leads to a decrease in the amplitudes of the off-diagonal peaks with temperature. The ground state is a symmetric wave function about the point $l=N/2$ in the $|l\rangle \equiv |l,N-l\rangle$ basis. At zero temperature, the density matrix has four equal peaks at four locations. The diagonal peaks in $\rho _{l,l^{\prime }}$ describe “populations”, whereas the off -diagonal peaks describe “coherences”. At finite temperatures, the density matrix acquires contributions from the excited states, most notably the antisymmetric first excited state. For *N* even, it is convenient to introduce the translated integer index $l_t\equiv l-N/2$. Antisymmetry implies that the off-diagonal coherence products $\Psi _{l_t}^{(1)}\Psi _{-l_t}^{(1)}$ are negative. These contribute to phase cancellation of positive ground state terms $\Psi _{l_t}^{(0)}\Psi _{-l_t}^{(0)}$. As a result, the thermal admixture of the antisymmetric first excited state contributes significantly to decoherence of the Cat states. We consider all exact excited states together with the ground state in our numerical calculations.

**Figure 7:**
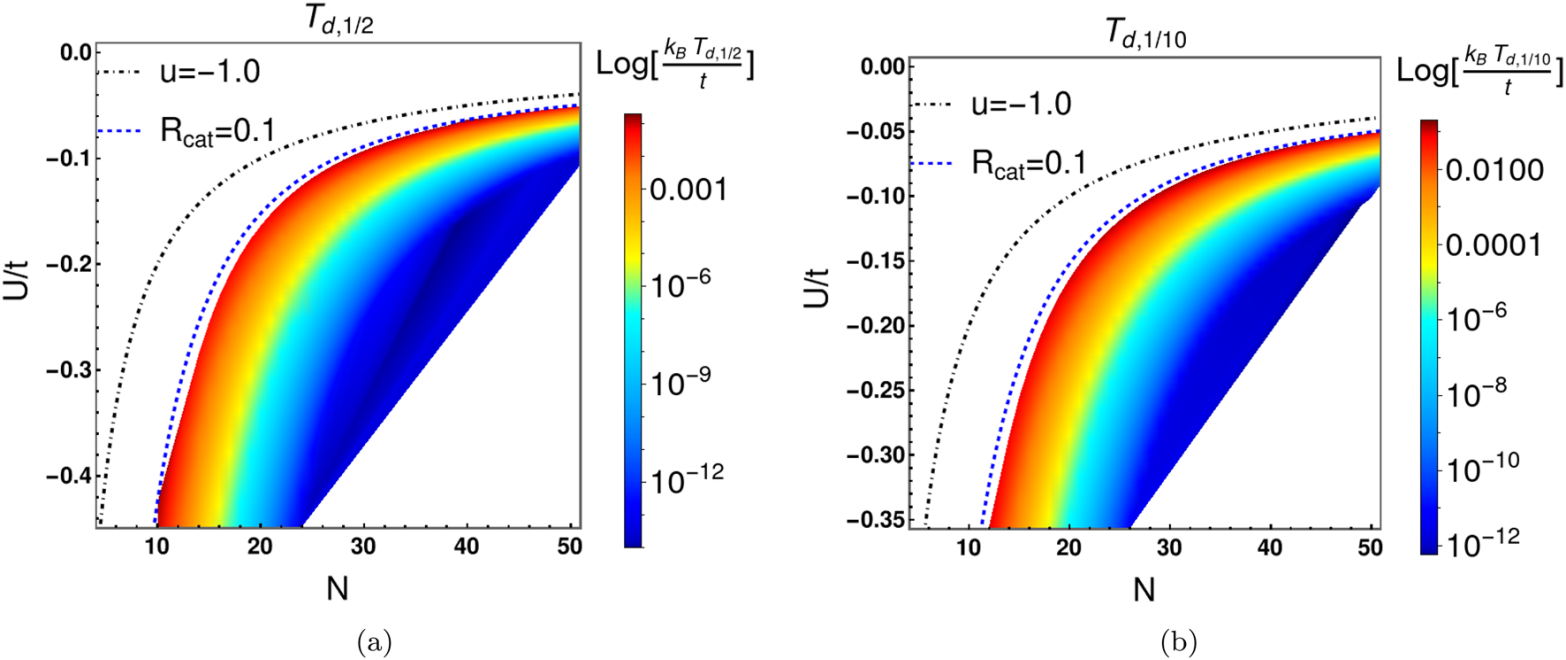
Decoherence temperatures ($T_{d,R}$) for Schrödinger Cat states in $N-U/t$ plane. Here $T_{d,R}$ is defined by the condition $\frac{\varvec{\rho }(T_{d,R})}{\varvec{\rho }(T=0)}|_{\text {off-diagonal-peak}}=R$. In panel (**a**), $R=1/2$ whereas in panel (**b**), $R=1/10$. The decoherence temperatures drop very rapidly as we move toward more well-developed Cat states. In panels (**a**) and (**b**), the blue dashed depicts the locus of points for which $R_{cat}=0.1$, where $R_{cat}\equiv \frac{|\Psi ^{0}(l_{t}=0)|^2}{|\Psi ^{0}( l_{\text {peak}})|^2}$. Decoherence temperatures are represented by the colour bar in $\log {[k_B T_{d,R}/t]}$ scale, where *t* is the hopping matrix elements between sites *a* and *b*. It is evident that Cat states are more robust and stable to small thermal fluctuations for large |*U*/*t*| and small *N*. This can be compared to the behaviour of $dE_{10}$ in Figs. 2c and 2d. For the same values of |*UN*/(2*t*)|, Cat states with large *N* and small |*U*/*t*| exhibit lower decoherence temperatures.

In the off-diagonal region of the ($l_t,l_t^{\prime }$) grid the antisymmetric excited state coherences cancel those of the symmetric states. Consequently, the amplitude of the off-diagonal peaks in the density matrix decrease rapidly with increasing temperature as shown in Fig. 6, panels a–d. We define the decoherence temperature ($T_{d,1/2}$) at which the amplitude of the off-diagonal peak is 1/2 times the amplitude of the diagonal peak at zero temperature. More generally, we define $T_{d,R}$ according to the condition that $\frac{\varvec{\rho }(T_{d,R})}{\varvec{\rho }(T=0)}|_{\text {off-diagonal-peak}} = R$. The ground state has two Gaussian peaks only if $u<-1$. Below this critical value, there are four peaks in the density matrix at zero temperature. However, the peaks are not well separated close to $u=-\,1.0$. We refer to this quantum state for which the Cat components are not well-developed as a weak Cat state. Panels e–h of figure (6) illustrate the behavior of the four peaks of the density matrix with increasing temperature for the weak Cat. In this case, it is very difficult to distinguish between the peaks.

We now focus on Cat states with well-separated components, and $R_{cat}\le 0.1$. Figure 7 illustrates the rapid lowering of the decoherence temperature as Cat states become better-developed and Cat components are well-separated. The color bars in Fig. 7 depict the decoherence temperatures $T_{d,1/2}$ and $T_{d,1/10}$ in $U/t-N$ plane. The blue dashed line represents the locus of points for which $\frac{|\Psi ^{0}(l_{t}=0)|^2}{|\Psi ^{0}( l_{\text {peak}})|^2}=R_{cat}$, takes on the value 0.1 in 7a and 7b. The decoherence temperature is always highest near blue dashed line but drops exponentially below it. Below the blue dashed line, Cat states are more well-defined with better separated components, but the separation $dE_{10}$ between the ground and first excited state becomes exponentially smaller. As a result the Cat states lose their coherence at exponentially low temperature.

Decoherence temperatures a listed in Tables 1 and 2 with the choice of $t=0.1$eV for $N=25$ and $N=50$, respectively. Clearly, decoherence occurs at lower temperature for larger values of |*U*/*t*| where the Cat states are more well-defined. For a given value of $u<-1.0$, the loss of coherence is even more severe for large *N* than for large |*U*/*t*|. The dynamical time scales describing decoherence and thermalization may be of interest in the future research^48^.

**Table 1:** Decoherence temperatures at $t=0.1$ eV ($\equiv 1162$ K) for $N=25$ bosons.

$\frac{UN}{2t}(\equiv u)$	*U* (meV)	$R_{cat}$	$T_{d, 1/10}$ (K)	$T_{d,1/2}$ (K)	$dE_{10}$ (K)
− 1.28	− 10.2	0.5	474	159	219
− 1.35	− 10.8	0.3	320	96.5	126
− 1.42	− 11.3	0.1	242	59.8	68.6
− 1.46	− 11.8	0.04	149	33.7	38.7
− 1.50	− 12.0	0.03	123	26.2	30.0
− 1.55	− 12.4	0.02	80.2	16.0	17.9
− 1.60	− 12.8	$8.0\times 10^{-3}$	48.8	9.3	10.6
− 1.65	− 13.2	$4.0\times 10^{-3}$	29.8	5.7	6.3
− 1.80	− 14.4	$1.0\times 10^{-3}$	6.4	1.2	1.3

Clearly, the decoherence temperatures closely track the energy separation $dE_{10}$ between the ground and first excited states. Increasing *u* values above $-\,1.28$ will increase both $R_{cat}$ and $T_{d,R}$ values. However, a large $R_{cat}>0.5$ indicates that the peaks of the density matrix and the components of the Cat state are not distinctly different.

**Table 2:** Decoherence temperatures at $t=0.1$ eV ($\equiv 1162$ K) for $N=50$ bosons.

$\frac{UN}{2t}(\equiv u)$	*U* (meV)	$R_{cat}$	$T_{d, 1/10}$ (K)	$T_{d,1/2}$ (K)	$dE_{10}$ (K)
− 1.18	− 4.7	0.45	363	118	156
− 1.20	− 4.8	0.28	286	87.1	108
− 1.25	− 5.0	0.09	116	38.8	45.6
− 1.30	− 5.2	0.03	71.5	14.9	16.9
− 1.45	− 5.8	$5.0\times 10^{-4}$	3.0	0.6	0.6
− 1.55	− 6.2	$4.1\times 10^{-5}$	0.3	$5.0\times 10^{-2}$	$5.2\times 10^{-2}$
− 1.75	− 7.0	$3.0\times 10^{-7}$	$3.3\times 10^{-2}$	$3.4\times 10^{-3}$	$5.8\times 10^{-4}$

The decoherence temperatures are considerably lower than for $N=25$ (Table 1).

## Particle number fluctuations

**Figure 8:**
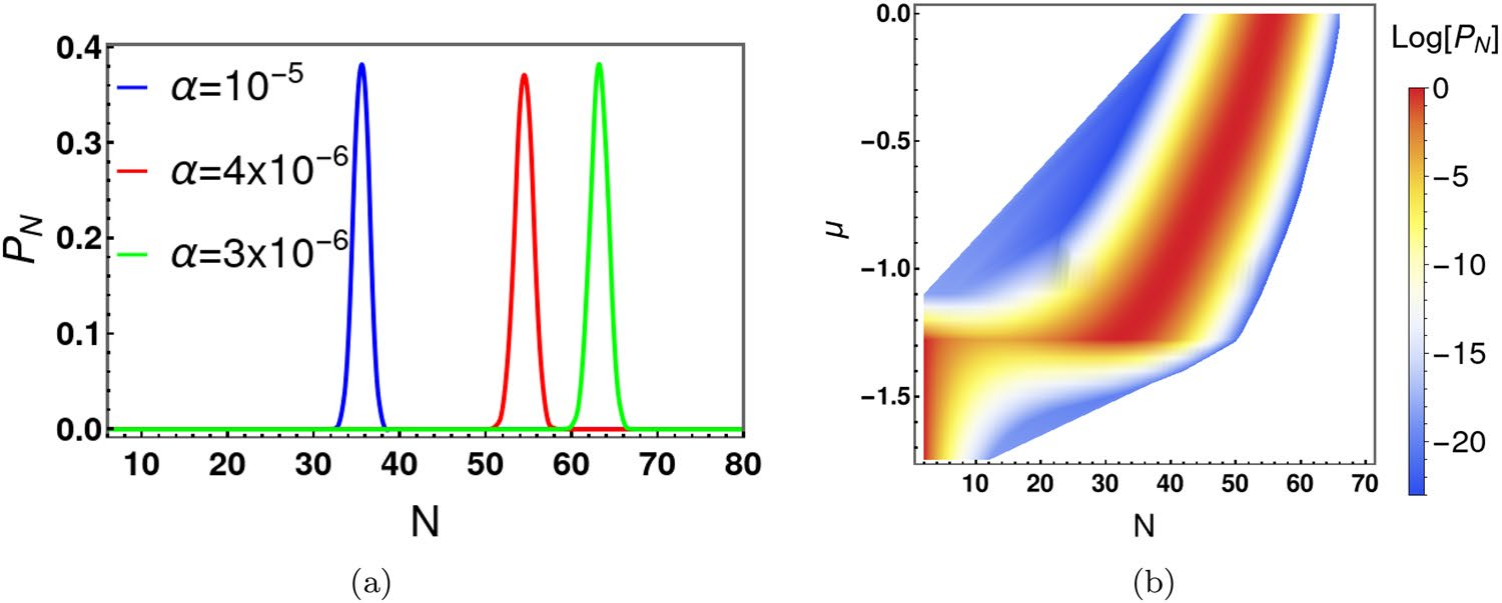
Panel (**a**) shows the particle number probability distribution for three different values of $$\alpha $$ at $$\gamma =4$$. $$P_N$$ is almost zero at large *N* for $$\alpha >0$$. The parameters used in our exact diagonalization of the many-body Hamiltonian and calculation of $$P_N$$ are $$U/t=-$$$$0.05,~\gamma =4,~ k_{B}T/t=0.1 $$ and $$\mu =-$$0.1. Panel (**b**) shows the distribution of $$P_N$$ in the $$\mu -N$$ plane. Here, $$P_N$$ is practically zero $$N >70$$. As a result, the grand partition sum is accurately described by keeping terms only up to $$N =70$$. The parameters used in panel (**b**) are $$U/t=-$$-$$0.05,~\gamma =4,~\alpha /t=4\times 10^{-6}$$ and $$ k_{B}T/t=0.01$$.

Another potential source of decoherence of the Schrödinger Cat state is through exchange of particles with a reservoir. We now discuss the two-site Bose-Hubbard model in the grand canonical ensemble. Here, the average particle number is determined by a chemical potential. The canonical partition function and probability for a particular N are given by: $Z_{N}=\sum _{i=0}^{N}\exp (-\frac{E_i -\mu N}{K_{b}T})$ and $P_{N}=Z_{N}/Z$, respectively. The grand partition function is $Z=\sum _{N}Z_{N}$.

The attractive Bose-Hubbard model (1) is physically unstable when placed in contact with a reservoir from which particles can enter the system. With a purely attractive, on-site Hubbard interaction, it is energetically favourable for an arbitrarily large number of particles to enter the system from the reservoir. This unmitigated accumulation of the particles is unphysical. In order the regulate this divergence, we introduce an additional repulsive term in the original Hamiltonian that is active when the particle number becomes very large. When *N* is not too large, the onsite attractive interaction remains dominant. The additional repulsive interaction is of the form ${\alpha (n_a+n_{b})^\gamma }$, where $\alpha$ is a positive energy parameter. Equation (2) in the $|l\rangle = |l,N-l\rangle$ basis, then becomes:

24 $$\begin{aligned} E\Psi _l=-t_{l-1}\Psi _{l-1}-t_{l}\Psi _{l+1}+ V_{l}\Psi _{l}+ {\alpha N^\gamma \Psi _{l}}. \end{aligned}$$

**Figure 9:**
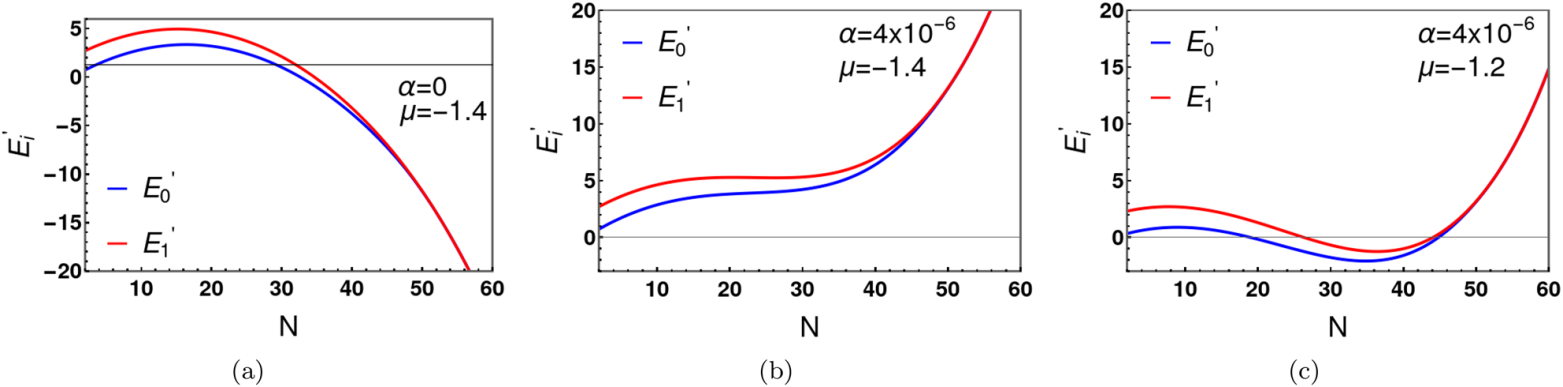
Ground and first excited state energies as a function of particle number for different choices of the repulsive energy coefficient $\alpha$. Panel (**a**) shows the (unphysical) instability of the ground state and first excited state energies in the absence of regulation ($\alpha =0$). Here energy decreases without bound for large *N*. For $\alpha >0$, (panels (**b**) and (**c**)), the probability, $P_N$, of *N* particles being admitted to the system from the reservoir tends to zero for large *N*. The energy eigenvalues have a global minimum at $N=0$ (panel (**b**)) for small $\mu$. But the global minimum shifts from $N=0$ to finite *N* above a critical value of $\mu$ (panel (**c**)). As a result, there is a sharp jump in $P_N$ at the critical chemical potential. The parameters used for this simulations are (**a**) $U=-0.05, ~t=1,~\mu =-1.4,~\alpha =0$, (**b**) $U=-0.05, ~t=1,~ \gamma =4, \alpha =4\times 10^{-6}, \mu =-1.4$ and (**c**) $U=-0.05, ~t=1,~ \gamma =4, \alpha =4\times 10^{-6}, \mu =-1.2$.

We numerically solve the above equation to determine all energy eigenvalues and eigenfunctions, for a large variety of choices of *N*. Figure 8a shows the behaviour of the probability distribution for different values of $\alpha$ at $\gamma =4$. Clearly, $P_N$ is zero in the large *N* limit and unlimited accumulation of particles from the reservoir is prevented. The value of *N* for which $P_N$ reaches its maximum is denoted as $N_{max}$. $N_{max}$ depends on all other variables $(\alpha , \gamma , U, t, \mu )$. We plot $P_N$, as function of the chemical potential $\mu$, in $\mu -N$ plane (see Fig. 8b). The sharp jump of the peak position ($N_{max}$) for specific $\mu$ values is straightforward to interpret. $P_N$ depends on the behaviour of the individual energy eigenvalues, $E_i$ with *N*. Figure 9 shows the behaviour of the ground and first excited state energies for different values of $\alpha$ and $\mu$. For $\alpha =0$, $E_{i}^{\prime } (\equiv E_{i}-\mu N)$ first increases with *N* and then decreases without bound (see Fig. 9a). As discussed, above, this situation is unphysical. With addition of the term $\alpha N^\gamma$ ($\alpha >0, \gamma =4$) to the Hamiltonian, the energy eigenvalues first increase and then possibly decrease with *N*, but eventually increase without bound for sufficiently large *N* as shown in Fig. 9b and c. Typically, $E_{i}^{\prime }$ has a local minimum near $N=0$, but a second deeper local minimum appears as a function of *N* for $\mu$ larger than a critical value (Fig. 9c). Accordingly, we see a sharp jump in $N_{max}$ as $\mu$ increases. The location of the jump depends on the values of $\alpha$ and $\gamma$. For example, when $\alpha =4\times 10^{-6}$ and $\gamma =4$, the critical chemical potential is $\mu =-1.25$.

**Figure 10:**
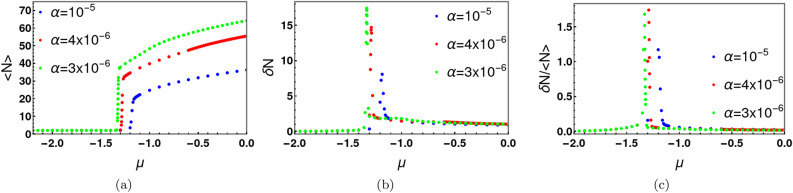
Panel (**a**) shows the sharp jump in the average boson number $$<N>$$ with $$\mu $$ in the grand canonical ensemble for three different values of the repulsion energy parameter $$\alpha $$ in the attractive Bose-Hubbard model with $$U/t=-0.05$$. For $$\alpha =4\times 10^{-6}$$, the system admits particles from the reservoir above the critical value of $$\mu =-1.25$$. The values of $$<N>$$ increase with the decrease of $$\alpha $$. In the canonical ensemble, Cat states are possible at $$T=0$$ for $$N>40$$. In the grand canonical ensemble $$<N><40$$ when $$\alpha =10^{-5}$$ for the range of $$\mu $$ shown. For $$\alpha =4\times 10^{-6}$$, $$<N>=55$$ at $$\mu =0$$. In this case a Cat type solution exist in the range $$-0.95\le \mu \le 0$$. The root mean square particle fluctuation $$\delta N$$ and $$\frac{\delta N}{<N>}$$ are depicted in panels (**b**) and (**c**) respectively. The parameters used in this simulations are $$U/t=-0.05,~\gamma =4 $$ and $$ k_{B}T/t=0.1$$.

The above analysis (see Fig. 8b) provides a convenient upper cutoff in particle number in our numerical simulations of the grand partition function for a given parameter range. For example, if $\alpha =4\times 10^{-6}$ and $\gamma =4$ the upper cutoff of particle number can be chosen as 70. This upper cutoff represents the minimum value of *N* in the grand partition sum above which our numerical results are independent of the truncation of the sum over *N*. We use this simplification to calculate the average number of particles ($<N>=\frac{1}{Z}\sum _{N}NZ_{N}$) and the root mean square of particle fluctuation ( $\delta N=\sqrt{<N^2>-<N>^2}$ ) in the grand canonical ensemble. Figure 10a shows the behaviour of average number of bosons as a function of $\mu$ at $U/t=-0.05$, for different values of $\alpha$. $<N>$ is zero for large negative values of $\mu$. As $\mu$ increases particles are eventually admitted into the system from the reservoir with a dramatic increase in $<N>$ at a critical $\mu$ value. $<N>$ continues to increase with further increase in $\mu$. The values of $<N>$ increase with the decrease of $\alpha$. For example, at $\mu =0$ and $U/t=-0.05$, $<N>=36$ for $\alpha =10^{-5}$ and $<N>=55$ for $\alpha =4\times 10^{-6}$. For the same *U*/*t*, the effective potential is a double well with two minima for $N>40$. The corresponding density matrix in the grand canonical ensemble exhibits four peaks when $<N>$ exceeds 40. For $\mu <0$ and $\alpha =10^{-5}$, Cat states do not exist. For $\alpha =4\times 10^{-6}$, Cat states exist for $-0.95\le \mu \le 0$. The root mean square fluctuation $\delta N$ is shown in Fig. 10b and $\frac{\delta N}{<N>}$ is depicted in Fig. 10c. As expected, $\delta N$ exhibits a sharp peak near the critical value of $\mu$. The density matrix in the grand canonical ensemble is defined as $\hat{\rho }^{g}(l,l^{\prime })=\sum _{N}P_{N}\hat{\rho }^{c}_{N}(l,l^{\prime })$, where $\hat{\rho }^{c}_{N}(l,l^{\prime })$ is the $N-$particle density matrix in canonical ensemble. Here we set $\hat{\rho }^{c}_{N}(l,l^{\prime })=0$ for any $l,l^{\prime }>N$. We calculate the decoherence temperatures in the grand canonical ensemble and compare them with those in the canonical ensemble. Table 3 lists the decoherence temperatures $T_{d,1/2}^{c}$ and $T_{d,1/2}^{g}$ in the canonical and grand canonical ensembles, respectively. The table shows decoherence temperatures in the canonical ensemble are slightly higher than those in the grand canonical ensemble. As expected, particle number fluctuations lead to further decoherence of the Cat states. However, thermal fluctuations are considerably more destructive to the coherence of the Schrödinger Cat.

**Table 3:** Particle number fluctuations are shown to cause decoherence over and above thermal fluctuations.

$\mu /t$	$<N>$	$T_{d,1/2}^{g}/t ~(\times 10^{-3})$	$T_{d,1/2}^{c}/t ~(\times 10^{-3})$ with $N=<N>$
		(Grand canonical)	(Canonical)
− 0.04	55.0	1.3	1.3
− 0.14	53.9	2.8	2.8
− 0.22	53.0	5.5	5.5
− 0.30	52.0	10.5	10.8
− 0.38	50.9	19.2	19.9
− 0.44	50.1	32.9	34.0
− 0.51	49.0	51.4	53.2
− 0.56	48.1	76.0	81.2

Here, $T_{d,1/2}^{g}$ and $T_{d,1/2}^{c}$ are the decoherence temperatures in grand canonical and canonical ensembles, respectively. These decoherence temperatures correspond to a factor of 2 decrease in the height of the off-diagonal peaks of the density matrix. The comparison to the canonical ensemble (final column) is made by choosing the particle number *N* equal to the average value $<N>$ in the grand canonical ensemble. The parameters used in the simulation are $U/t=-0.05, ~\alpha /t=4\times 10^{-6},$ and $\gamma =4$.

## Discussion and conclusions

In summary, we have shown that Schrödinger Cat states of the two-site Bose-Hubbard model are highly susceptible to loss of quantum coherence when placed in contact with a physical environment. In general, the more distinguished the components of the Cat state, the more unstable it is to decoherence. In the model we studied, this arises from the near degeneracy of the symmetric and antisymmetric quantum superpositions of the Cat components. This illustrates the elusive nature of stable quantum entanglement involving large numbers of material particles.

One possible realization of Schrödinger Cat states is in ultracold atomic systems in an external magnetic field, in which the Feshbach resonances can be utilized to control the attractive interaction between atoms^20,49^. A bosonic atom may be trapped spatially in nearby minima of a shallow optical trap that allows tunnelling between the local minima. This would require nano-Kelvin temperature scales. Another interesting possibility is in semiconductor quantum wells containing stable, bound electron-hole pairs. These bosonic excitations can couple strongly to optical cavity modes forming exciton-polaritons that have effective masses that are $10^{-4}$ to $10^{-5}$ times the bare electron mass. In the context of photonic crystal cavity modes, it has been suggested that these exciton-polaritons could exhibit Bose-Einstein condensation at room temperature^50,51^. When the relevant semiconductor quantum well is sandwiched by 3D photonic band gap materials, above and below, there are degenerate valleys in momentum space where the exciton polaritons may condense. In exciton systems, Feshbach resonances can occur without recourse to an external magnetic field^24^, enabling attractive interactions between the exciton-polaritons. Hopping between the degenerate valleys in the momentum space may be facilitated by interactions with phonons in the quantum well.

The realization of stable quantum superposition states of material particles on a mesoscopic scale would be a significant advance in quantum science. We hope that our analysis provides useful insights into the fundamental challenges involved and the reasons why such quantum superpositions are particularly delicate.

## Data Availability

All data generated or analysed during this study are included in this published article.
